# Modal properties of fruit-rachilla system of the macaw palm

**DOI:** 10.1371/journal.pone.0237291

**Published:** 2021-01-25

**Authors:** Flora Maria de Melo Villar, Francisco de Assis de Carvalho Pinto, Fabio Lucio Santos, Daniel Marçal de Queiroz, Mariana Ribeiro Pereira, Domingos Sárvio Magalhães Valente

**Affiliations:** 1 Departamento de Engenharia Agrícola, Mecanização Agrícola, Universidade Federal de Viçosa, Viçosa-MG, Brasil; 2 Departamento de Engenharia, Engenharia Mecânica, Universidade Federal de Lavras, Lavras—MG, Brasil; Univerza v Mariboru, SLOVENIA

## Abstract

The macaw palm has been domesticated due to its potential use in the production of biofuel, in addition to several co-products that can be generated from its oil and pulp. One of the current challenges in this area is the harvesting, as there are no specific machines for this operation. Therefore, it is necessary to determine the appropriate information regarding the physical properties of the plant, so that it is feasible to develop the technologies necessary for the commercial scale application of macaw palm, allowing it to contribute to the sustainable production of raw material for the biofuel industry and other co-products. The principle of mechanical vibration can be used to shed fruit from trees when ripe, and it can be a method used for harvesting. Thus, as proposed in this study, it was necessary to study the dynamic behavior of the fruit-rachilla system during vibration. Hence, the modal properties of the system were determined. A study on the dynamic behaviors was carried out using a deterministic finite element model, and the natural frequencies were obtained through a frequency-scanning test to evaluate the model. The mean relative error (MRE) between the measured and simulated natural frequencies was also used to evaluate the model. The natural frequencies, determined experimentally, varied from 26.21 to 33.45 Hz on average, whereas the simulated frequencies varied from 24.81 to 39.27 Hz. The overall MRE was 9.08%. Once the model was validated, a sensibility test was carried out, which showed that the density of fruit and the elasticity modulus are the parameters that most influence the natural frequencies of the fruit-rachilla system.

## Introduction

There are many studies, even if they are just beginning, about oil quality, genetic improvement, taxonomy of the palm popularly known as macaw palm. The macaw palm is a promising crop as a feedstock for biofuels owing to its high oil yield and quality; however, it is still employed using an extractive system. One of the main challenges in the Brazilian agro-energy sector is related to the generation of information and technology to develop a sustainable source of feedstock for the production of biofuels [1, 2]. The use of new technologies for fruit harvesting and processing is essential to allow the macaw palm to become commercially profitable to farmers, and to facilitate intensive exploitation. Nowadays, the fruits are allowed to ripen and come off the bunch falling to the ground.

The oil quality can be affected if the fruit is not harvested and processed within the appropriate maturation period, however, there are still no specific machines for harvesting the fruits, once the macaw palm culture is still in the domestication process. According to [3] was observed that the acidity level and oxidative stability of macaw oil are affected by the age of the fruit and its storage period.

Mechanical vibrations can be used as an option for the process of removing the fruit from the rachilla, either in a biofuel plant or during harvesting. Like other fruits, such as coffee, olive and citrus [4–6] etc., the modal properties, natural frequencies, and modes of vibration of the fruit-rachilla system are the basic data required to develop equipment employing mechanical vibrations to harvesting.

Because the macaw palm domestication process started recently, a natural and significant variety is present among the different accessions, which generates fruits of different sizes and maturation. To help in the development of machines used to harvest the fruit, it is a general practice to generate a mathematical system model, in order to obtain information about its dynamic behavior when subjected to mechanical vibrations. [7] Concluded that the simulation can be useful for studying the frequencies for detachment fruits under different conditions and this information can be useful to help in the development of harvesters and improvement of their performance. Once we have a proper model, we can simulate the dynamic behavior of the fruit-rachilla system in order to estimate the modal properties.

The finite element method can be used to solve different problems by dividing the model into the specific elements of the body being studied and generating a mesh as well as a group of equations that describe the behavior of the variables involved [8]. [9] Used a model to evaluate the dynamic behavior of the coffee tree using techniques of the finite element method. From this technique, it was possible to determine natural frequencies related to the different modes of vibration. In another case of coffee, a mathematical simulation using the finite element method was applied to obtain information regarding the modal parameters of the fruit-peduncle [4].

The objective of this work was to develop a model to describe the dynamic behavior of the macaw palm fruit-rachilla system, and to simulate this system in order to identify which parameter has the greatest influence on its natural frequencies.

## Material and methods

The fruit bunches used to determine the modal properties of the fruit-rachilla system were collected at UFV’s Active Germplasm Bank (AGB), Araponga city, Minas Gerais state, Brazil. Tests were conducted using bunches with green fruits. The harvesting of the fruit bunches was performed on Sept. 30, 2015, and the tests were carried out on the same date. Four palm trees of different accessions were used and identified as BD 27 (from the Abaeté region, Minas Gerais state), BD 40 (from the Pitangui–Martinho Campos region, Minas Gerais state), BGP 29 (from the Prudente de Morais–Matozinhos region, Minas Gerais state), and BGP 35 (from the Mirandópolis region, São Paulo state).

The mechanical, physical, and geometric properties were used as the input parameters during the computational modeling and simulation of the fruit-rachilla system to determine the natural frequencies and vibration modes (Table 1). The mechanical properties of the fruits, the elasticity modulus, and the Poisson coefficient were assumed to be equal to those of the rachilla. [10] Conducted the modal analysis and the natural frequency for orange using finite element method, validated by experimental measurements. For this purpose, among other properties, the physical-mechanical properties of the modulus of elasticity and Poisson's ratio were used, which were compared for green and ripe fruits in relation to the natural frequency. The natural frequencies of the fruit-rachilla (eigenvalues), as well as the respective vibration methods (eigenvectors), were obtained through the formulation and calculation of the eigenvalue and eigenvector problems. For the modeling, systems with multiple degrees of freedom were considered, represented by a system of differential equations. The analysis of the dynamic behaviors was carried out using finite element discretization. The geometry discretization, modeling, and visualization of the results were conducted using the software Autodesk® Fusion 360®, student version.

**Table 1:** Mechanical, physical, and geometric properties used as input parameters during the computational modeling and simulation of the fruit-rachilla system for the accessions BD 27, BD 40, BGP 29, and BGP 35.

	BD27	BD 40	BGP 29	BGP 35
**d_r_ (mm)**	3.73	3.29	3.61	4.34
**ρ_r_ (g cm^-3^)**	0.63	0.47	0.62	0.88
**a (mm)**	32.44	45.24	58.09	39.91
**b (mm)**	39.59	44.13	47.41	41.55
**c (mm)**	38.80	43.71	47.12	41.29
**ρ_f_ (g cm^-3^)**	1.05	1.15	1.08	1.12
**E (MPa)**	238.90	260.70	293.90	194.00
**ν**	0.37	0.37	0.37	0.37

d_r_, rachilla diameter; ρ_r_, rachilla density; a, the largest characteristic dimension of the fruit; b, intermediate characteristic dimension of the fruit; c, the lowest characteristic dimension of the fruit; ρ_f_, fruit density; E, rachilla elasticity modulus, and ν, rachilla Poisson coefficient [11, 12].

In the geometry discretization step, a convergence test was conducted to evaluate the type of mesh and the most adequate average element size. The convergence test yielded 10-node tetrahedral elements with an average side length of 0.001 m.

In the modeling step, three-dimensional models of the fruit-rachilla system were elaborated using the software Autodesk® Fusion 360®. The model was made up of a rachilla crimped at one of its extremities. The rachilla length was standardized as 12 cm. To simulate the system, the fruits were assumed to be attached at a position three-quarters of the rachilla length from the crimped side. The boundary conditions were also defined during the modeling step. Once the system modeling was defined, the Lanczos algorithm in the Autodesk® Fusion 360® software was used to determine the eigenvalues and eigenvectors. Using the Lanczos algorithm, the model's recursions were developed using a vector block instead of a simple vector. To extract the number of eigenvalues required, this method employs an automated exchange strategy combined with the Sturm sequence check [13].

**Fig 1:**
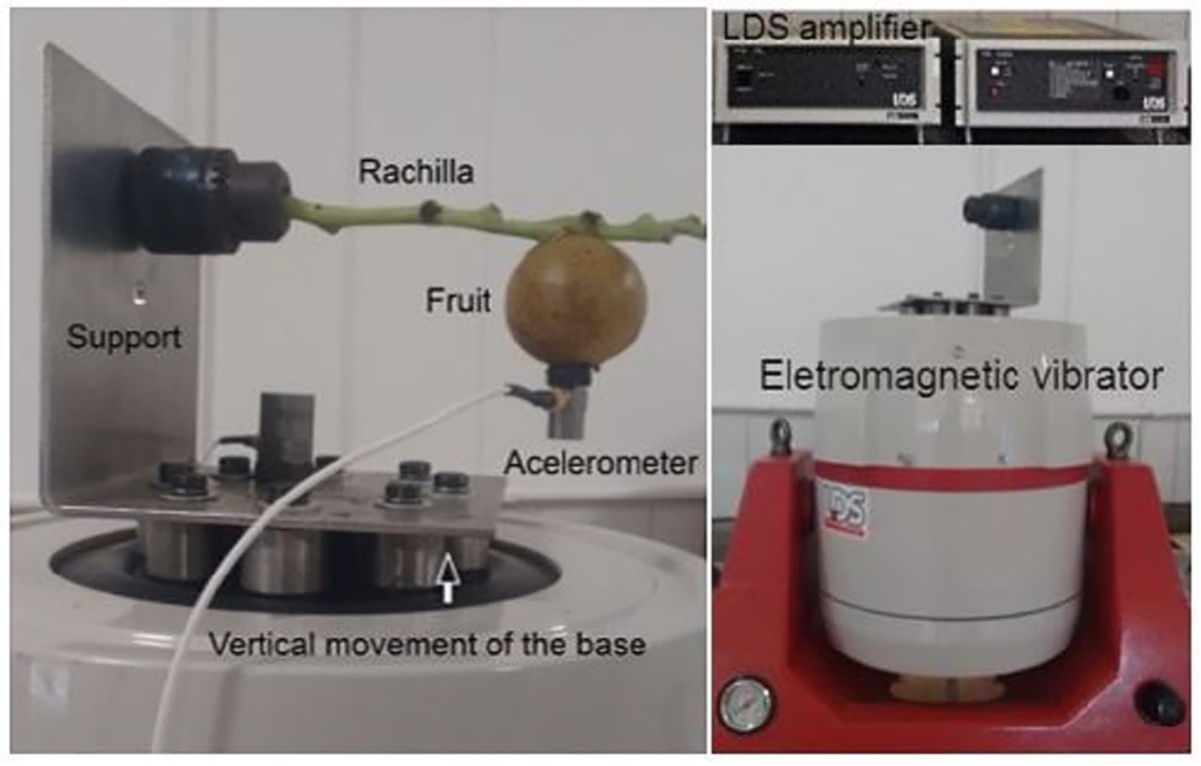


Before the simulation, the model was validated using the natural frequencies acquired through the experiment process. A scanning test was conducted using ten samples from the fruit-rachilla system for each accession. To promote the excitation of the fruit-rachilla system, a Ling Dynamic Systems (LDS) instrument was used (Fig 1). The system was composed of a Dactron COMET_USB_ signal generator, an LDS PA 1000L amplifier, and an LDS V-555 electromagnetic vibrator. The scanning test was performed by increasing the frequency progressively by 1.5 octaves/min^-1^, from 10 to 40 Hz, with a peak-to-peak displacement amplitude of 0.5 mm. The system was excited with an acceleration rate 15-times the gravitational acceleration, and sampling at a rate of 500 Hz.

The fruit-rachilla samples were clamped at 2 cm lengthwise by using a fixed support in the vibrator, using the same length as in the geometric model. The sample fruits were attached to the rachilla at nearly the same place as for the geometric model. To acquire the data, a piezoelectric accelerometer LW174002 with a sensibility of 100.7 mVg^-1^, and an acquisition modulus cDAQ-9174 from National Instruments were used. The accelerometer was fixed to the fruit for measuring the acceleration of the system in the vertical direction. The accelerometer mass was 5.6 g, which is 15% less than that of the rachilla-fruit mass.

The acquired data were transformed into the frequency domain using a Fourier transform to find the resonance frequency that occurred at the peak displacement amplitude. This resonance occurred when the system excited frequency was equal to the fundamental frequency. To evaluate the models, the relative error between the measured and simulated fundamental frequencies was determined (Eq 1).

$$MRE\;(\%)=\frac{\hat{y}-y}{y}100$$ (1)

where,

MRE–mean relative error;

*y_i_*–measured first natural frequency, Hz; and

${\hat{y}}_{i}$–simulated first natural frequency, Hz.

A fundamental frequency was simulated using the average of the modulus of elasticity, fruit and rachilla density for all accessions. To identify which parameter had the greatest influence on fundamental frequencies a sensitivity analysis was carried out. The rachilla’s modulus of elasticity and the fruit, and the rachilla density, were varied by ± 20% on sensitivity analysis. Therefore, six fundamental frequencies were obtained and compared to the average fundamental frequency.

## Results and discussion

**Fig 2:**
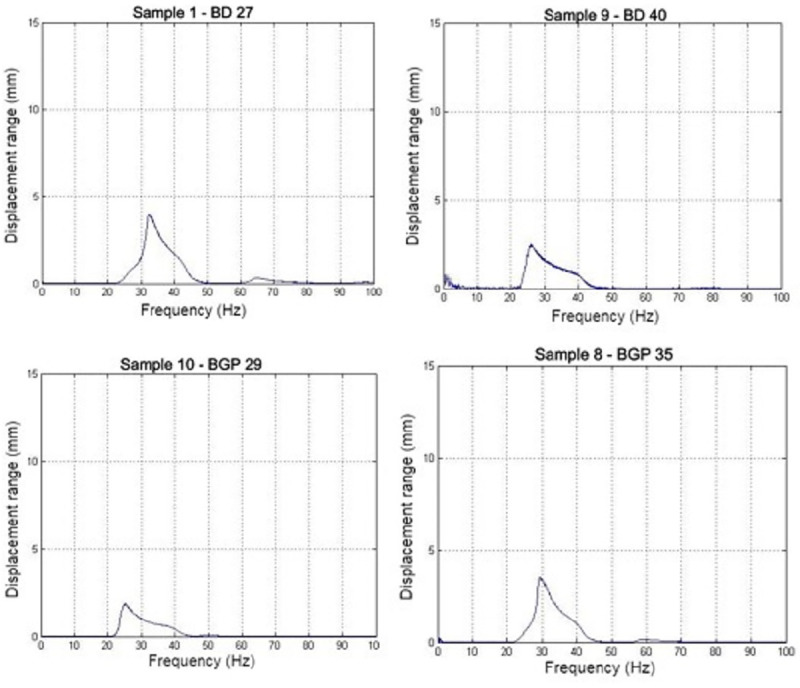


The first natural frequencies were associated with resonance frequency. The resonance frequency was identified when occurred a peak amplitude displacement in frequency sampled range (Fig 2). The results presented in Table 2 the mean relative errors and the descriptive statistical analysis of the first natural frequencies of each accession achieved experimentally through the scanning test and mathematically through the simulation. Some of the fruit-rachilla systems were eliminated because they presented frequencies above the pre-determined maximum limit of 40 Hz, which was attributed to errors occurring during the laboratory test.

**Table 2:** Descriptive statistics for the measured and simulated natural frequencies of the fruit-rachilla systems for the BD 27, BD 40, BGP 29, and e BGP 35 plants, as well as the mean relative errors between the measured and simulated values.

Plant accession	First Natural Frequency	N	Mean (Hz)	Standard deviation (Hz)	CV (%)	MRE (%)
BD 27	Measured	9	30.51 24.81	4.64 1.33	15.20 5.37	-14.95
	Simulated					
BD 40	Measured	10	26.1 32.40	4.15 1.48	15.82 4.57	30.71
	Simulated					
BGP 29	Measured	10	29.96 39.27	2.75 5.94	9.17 15.13	30.89
	Simulated					
BGP 35	Measured	8	33.45 26.50	4.16 4.98	12.44 18.77	-18.23
	Simulated					
All	Measured	37	29.37 31.13	4.51 5.84	15.34 18.77	9.08
	Simulated					

n, number of samples; CV, coefficient of variation; MRE, mean relative error.

**Fig 3:**
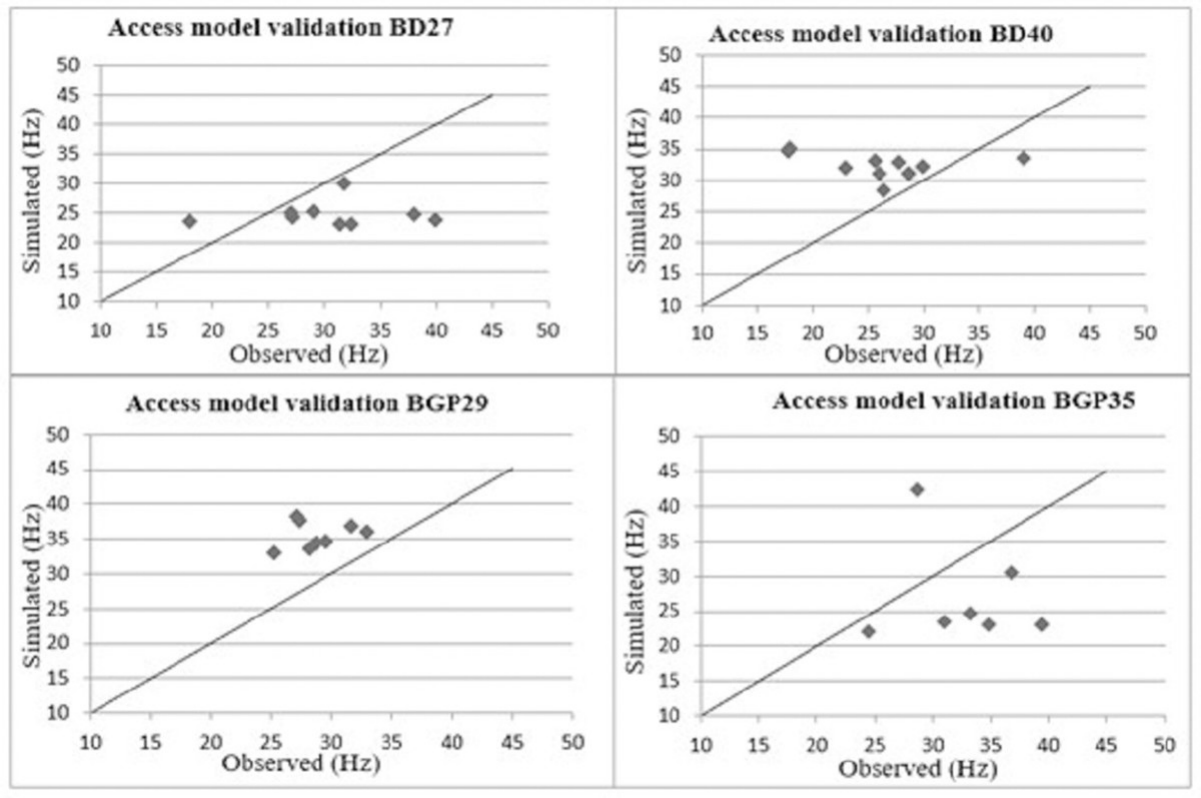


In Fig 3 shows the model's validation in graphic form to predict the fundamental frequency of the accessions studied, comparing values observed experimentally with the simulated values.

The natural frequencies obtained through the scanning test varied from 26.21 to 33.45 Hz on average with the coefficient of variation considered to be either low (between 0% to 10%) or medium (between 10% to 20%) for agricultural products [14]. The natural frequencies obtained through the mathematical simulation varied on average from 24.81 to 39.27 Hz with the coefficient of variation also considered as either low or medium for agricultural products. When analyzing the MRE of each plant accession, we observed that the proposed mathematical model presented a significant variation in estimating the natural frequencies with an MRE ranging from -14.95 to 30.89%.

The best result obtained during the simulation was for accession BD 27 with an MRE equal to -14.95%. When the MRE is closer to zero, the model’s capacity to estimate the natural frequencies is better accurate. The worst result was for accession BGP 29 with an MRE equal to 30.89%. The model underestimated the fundamental frequency in two plants accessions (negative MRE) and overestimated (positive MRE) in two other plants accessions. Thus, the model did not show trend by underestimating or overestimating the experimental data. This behavior, most likely, happens due to mechanical and geometrical properties variation between plants of different accessions.

[15] Conducted a modal analysis for the fruit-peduncle system of a coffee plant using the finite element method, compared the results with an analytical solution, and achieved a maximum MRE of 1.88%. In the present study, an analytical solution was not obtained. However, a comparison of the values that were obtained experimentally, with values obtained by simulation was carried out. The determined MRE can be explained through the approaches and considerations applied to enable the simulation.

[9] Found natural frequencies for coffee between 10 and 30 Hz in laboratory tests and concluded that this information may be useful for the design of machines that use mechanical vibrations to detachment the fruits. In addition, the authors found a linear correlation among the values for natural frequencies obtained by numerical simulation and laboratory tests, validating the model to predict the behavior of the coffee tree when submitted to mechanical vibrations.

[16] Used the principle of mechanical vibration to harvest macaw palm fruit and concluded that this technique is applicable and promising. A prototype was used to harvest the fruits with an average operational efficiency of more than 90%; however, no previous analysis of the modal properties was carried out, which could improve the performance of the cutting machine.

**Fig 4:**
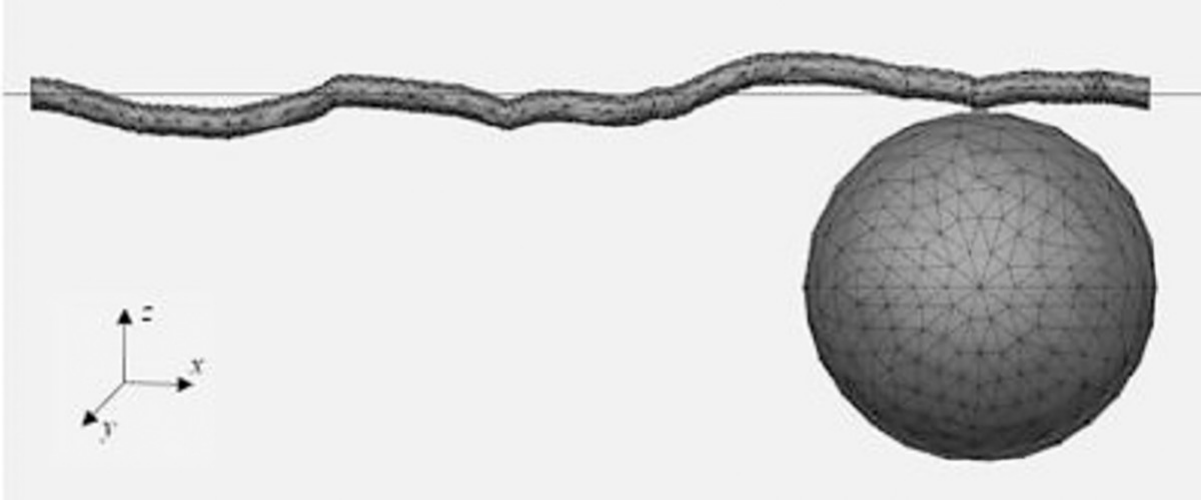


Because the accelerometer was positioned only for measurements in the z-direction, the modes of vibration obtained through the simulation for all plant accessions correspond to the vertical vibrations (Fig 4).

The elasticity modulus of the fruits, and the ratio Poisson, used as input parameters during the simulation, were the same as those applied for the rachilla, and the rachilla was considered with a unique diameter. These considerations may have influenced the model results because the actual rachilla diameter is not constant, and the fruits likely present mechanical properties differing from those of the rachilla.

The first natural frequencies were influenced by the elasticity modulus and Poisson’s ratio more significantly when compared to the density (Table 3). When Poisson’s ratio and elasticity modulus were decreased by 20%, the fundamental frequency was increased by 1.87% and 3.47% on average, respectively. When Poisson’s ratio and elasticity modulus were increased by 20%, the fundamental frequency was decreased by 1.36% and 4.71% on average, respectively.

**Table 3:** Average simulated natural frequencies during the sensibility analysis when the rachilla density, elasticity modulus, and Poisson coefficient were varied by ±20%, and the differences between the average simulated natural frequency of the model (as a percentage).

Average		Simulated Natural Frequency (Hz)
		31.13
E	-20%	29.66 (- 4.71%)
	+20%	32.21 (+ 3.47%)
Υ	-20%	30.71 (- 1.36%)
	+20%	31.71 (+ 1.87%)
Ρ	-20%	31.17 (+ 0.14%)
	+20%	30.97 (- 0.53%)

E, elasticity modulus; υ, Poisson coefficient; ρ, rachilla density.

The density property showed to have the lowest effect on the first natural frequency. The first natural frequency increased 0.14% when the density was decreased by 20% and decreased 0.53% when the density was increased by 20%. The natural frequency is directly proportional to the hardness, as determined based on the geometry and elasticity modulus, and inversely proportional to the mass, which is related to the density [17]. Therefore, when an increase in the elasticity modulus or a decrease in the density occurs, the natural frequency is higher, as it was seen in the analyzed case, showing a positive difference.

The model used for the simulation considered only the rachilla density, because the elasticity modulus of the fruits was not determined. So, these results showed low density influence on the model. However, if the model were more detailed and it was possible to have a fruit elasticity modulus, the density would have more influence on the natural frequency. But still, it would show the same behavior.

## Conclusion

A model was developed to estimate the modal properties of the macaw palm fruit-rachilla system (natural frequencies and modes of vibration). For the experimental data, the average fundamental frequency found was 29.37 Hz. As for the simulated data, the average fundamental frequency found was 31.13 Hz. The average relative error of the model was 9.08%. The closer to zero, the better the model's ability to estimate the natural frequency of the system, therefore, we can consider that the model was able to predict satisfactorily the dynamic behavior of the fruit-rachilla system, in view of the existing variability between accessions.

The fundamental frequencies found are associated with the vertical vibration mode.

From the sensitivity analysis, it was possible to identify that the parameters that most influence the estimation of natural frequencies are the elasticity module and the density of the fruits.

This study sought to obtain information to assist in the design of machines for the harvest of macaw palm by mechanical vibrations, since this is a culture in the process of domestication and still needs a lot of information to find the best way, still under study, for developing machines for both harvesting and fruit processing.

## Supporting information

S1 Data (XLSX)

S2 Data (XLSX)
